# Serum Vitamin D Levels Over Time and the Incidence of Atrial Fibrillation in the HUNT Study

**DOI:** 10.1210/jendso/bvaf168

**Published:** 2025-11-05

**Authors:** Lin Jiang, Yi-Qian Sun, Vegard Malmo, Xiao-Mei Mai

**Affiliations:** Department of Public Health and Nursing, Faculty of Medicine and Health Sciences, Norwegian University of Science and Technology, Trondheim N-7491, Norway; Clinic of Cardiology, St. Olav's Hospital, Trondheim 7030, Norway; Department of Clinical and Molecular Medicine, Faculty of Medicine and Health Sciences, Norwegian University of Science and Technology, Trondheim N-7491, Norway; Department of Pathology, Clinic of Laboratory Medicine, St. Olav's Hospital, Trondheim University Hospital, Trondheim 7030, Norway; TkMidt-Centre for Oral Health Services and Research, Trondheim 7030, Norway; Clinic of Cardiology, St. Olav's Hospital, Trondheim 7030, Norway; Department of Circulation and Medical Imaging, Norwegian University of Science and Technology, Trondheim N-7491, Norway; Department of Public Health and Nursing, Faculty of Medicine and Health Sciences, Norwegian University of Science and Technology, Trondheim N-7491, Norway

**Keywords:** serum 25-hydroxyvitamin D, incidence of atrial fibrillation, the HUNT Study, repeated measurement, Mendelian randomization

## Abstract

**Background:**

Evidence of the association between serum vitamin D levels and atrial fibrillation (AF) is inconclusive. Thus, this study investigated the relationship between long-term average serum 25-hydroxyvitamin D [25(OH)D] levels and AF incidence in the Norwegian Trøndelag Health (HUNT) Study using a prospective cohort design and a Mendelian randomization (MR) approach.

**Methods:**

A total of 3394 adults with 2 measurements of serum 25(OH)D at HUNT2 (1995-1997) and HUNT3 (2006-2008) and without AF at HUNT3 were followed up to 2021. Average serum 25(OH)D levels over 10 years were categorized into <50 and ≥50 nmol/L. AF diagnoses were retrieved from hospital registers and validated by doctors. Cox regression was used to calculate hazard ratios (HRs) and 95% confidence intervals (CIs). Furthermore, a 1-sample MR was conducted among 36 554 adults who participated in both HUNT2 and HUNT3 using the Wald ratio method.

**Results:**

During a median 12-year follow-up, 304 AF cases were diagnosed. Serum 25(OH)D levels <50.0 nmol/L were associated with a 27% reduced incidence of AF (HR 0.73, 95% CI 0.57-0.93) compared with ≥50 nmol/L after adjustment for confounders. A genetically determined 10 nmol/L decrease in the serum 25(OH)D levels was associated with a 7% reduced incidence of AF (HR 0.93, 95% CI 0.86-1.00) in the 1-sample MR. Sensitivity analyses supported this association.

**Conclusion:**

Using both traditional observational and 1-sample MR approaches, the study suggested a consistently positive association between long-term average serum 25(OH)D levels and incidence of AF in the Norwegian HUNT population.

## Background

Atrial fibrillation (AF), the most commonly diagnosed arrhythmia affecting over 60 million people worldwide [1], can increase the risk of stroke, myocardial infarction, and sudden cardiac death [2]. The prevalence of AF increases with age [1]. With the global aging population, it is important to identify modifiable risk factors [1]. Although the pathogenesis of AF is not fully understood, structural and electrical remodeling, regulation of the renin-angiotensin-aldosterone system, inflammation, and endothelial function are suggested critical elements in the development of AF [1, 3].

Vitamin D is a nutrient made in the skin from sunlight or obtained from foods like fatty fish and supplements like cod liver oil [4]. Serum 25-hydroxyvitamin D (25(OH)D) levels indicate total vitamin D status, reflecting sunlight exposure, diet, and supplements [5]. Vitamin D insufficiency, typically assessed by a serum 25(OH)D concentration less than 50 nmol/L, affects over 50% of the world's population, particularly in winter [6, 7].

Serum 25(OH)D seems to play a role in various physiological processes such as regulation of the aforementioned renin-angiotensin-aldosterone system and inflammation [8, 9]. Therefore, it may be linked to the development of AF, but most previous prospective studies have not reported any associations [10, 11]. Since vitamin D levels fluctuate over time, a single measurement may not accurately represent a person's long-term vitamin D status [12]. As a result, a single measurement may lead to random measurement error and regression dilution bias in the earlier studies [13, 14]. More prospective studies that incorporate repeated measurements of serum 25(OH)D are needed.

During the past decade, the Mendelian randomization (MR) method has been widely used to assist in drawing causal inference. The MR approach mimics a randomized controlled trial (RCT) in an observational setting by using genetic variants as instrumental variables for the risk factor of interest [15]. Since genetic variants are randomly assigned at conception and remain stable over the lifetime, bias due to reverse causation may be avoided and the influence of residual confounding is reduced [16]. It can offer supplementary evidence for causal relationships, while being less expensive and less time-consuming compared to RCTs [15]. There is only 1 well-conducted 2-sample MR study on this topic, which has not found any association [17]. Compared with 2-sample MR, 1-sample MR benefits from better control of confounders and reduced bias from population heterogeneity by utilizing individual-level data from a single population [15]. To our knowledge, no 1-sample MR has been conducted.

Hence, we aimed to examine the relationship of average serum 25(OH)D levels over 10 years with the incidence of AF in a prospective cohort study using the Norwegian Trøndelag Health (HUNT) population. Furthermore, we conducted a 1-sample MR to assess the causal effect of serum 25(OH)D on AF incidence.

## Methods

### Study Design and Population

The HUNT Study is a large population-based health study that has been carried out in 4 phases: HUNT1 (1984-86), HUNT2 (1995-97), HUNT3 (2006-2008), and HUNT4 (2017-2019) in Trøndelag County, Norway [18, 19]. All adults over 19 years old were invited to complete general questionnaires on health and lifestyle status and undergo clinical examinations [18, 19]. In addition, information on deaths and emigration was regularly updated from the Norwegian National Registry [18, 19].

**Figure 1.**
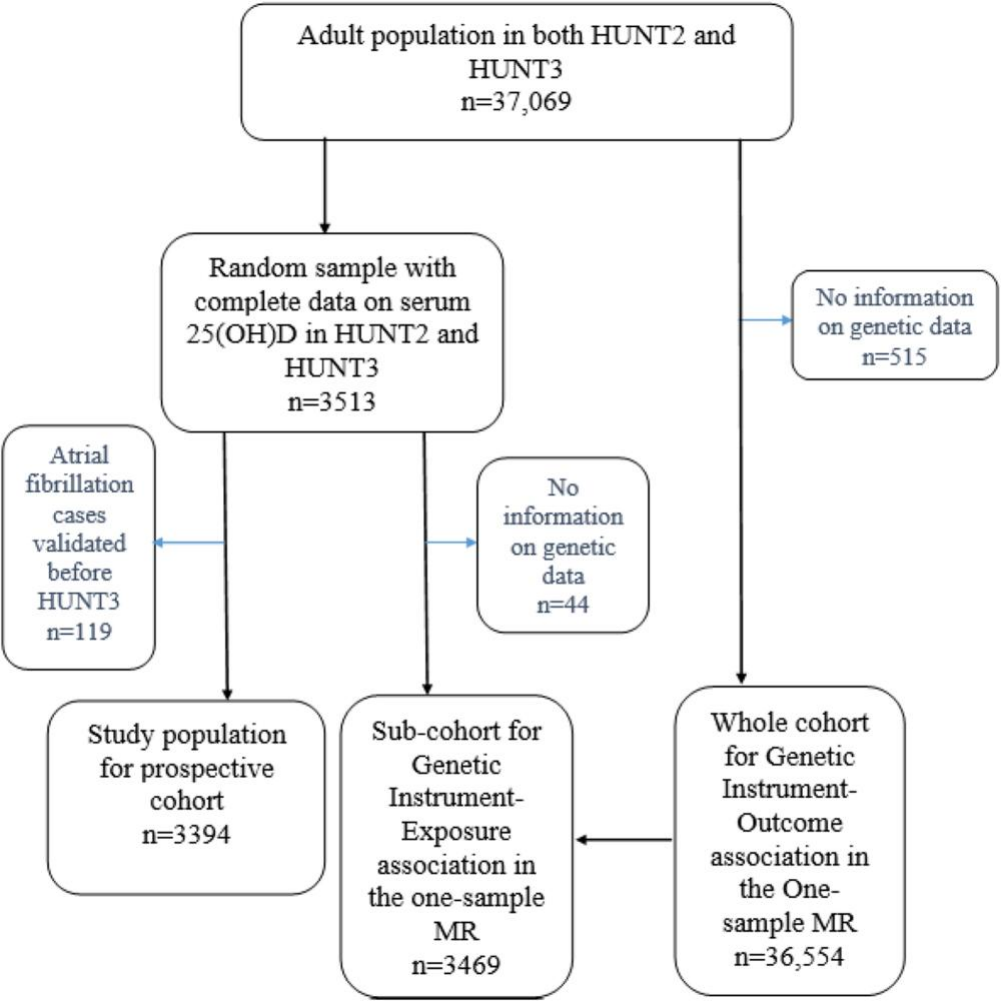
Flow chart of study populations. Abbreviations: 25(OH)D, 25-hydroxyvitamin D; HUNT, Trøndelag Health Study; MR, Mendelian randomization.

For our study, we included 37 069 adults who participated in both HUNT2 and HUNT3 (Fig. 1). In the prospective cohort study, a random sample of the adult population with complete data on serum 25(OH)D levels in both HUNT2 and HUNT3 were included (n = 3513). We followed them from HUNT3 (baseline) until the date of AF diagnosis, death, emigration, or the end of follow-up (January 31, 2021), whichever occurred first. After excluding participants with a validated AF diagnosis before HUNT3 (n = 119), 3394 participants were left in the prospective cohort study.

Of the 3513 individuals with complete data on serum 25(OH)D levels in both HUNT2 and HUNT3, 3469 had information on genetic data and were included as a subcohort in the 1-sample MR to estimate the genetic instrument-exposure association. The cohort used for estimating the genetic instrument-outcome association (n = 36 554) consisted of participants with complete genetic data derived from the 37 069 adults who participated in both HUNT2 and HUNT3 (Fig. 1).

### Measurements and Standardization of Serum 25(OH)D Levels

Serum 25(OH)D levels in HUNT2 and HUNT3 were measured at the HUNT Biobank using the LIAISON 25-OH Vitamin D TOTAL (DiaSorin, Saluggia, Italy; RRID: AB_2811287, https://scicrunch.org/resolver/AB_2811287), a fully automated, antibody-based, chemiluminescence assay [20]. The detection range of the assay for serum 25(OH)D is 10 to 375 nmol/L. Serum 25(OH)D levels were standardized to reflect October values for each individual, accounting for seasonal changes due to the high latitude of Norway [21]. The long-term average serum 25(OH)D levels were calculated as the mean of the 2 measurements in HUNT2 and HUNT3. For simplicity, the average season-standardized levels over 10 years are termed the serum 25(OH)D levels thereafter in this study. We categorized serum 25(OH)D levels into 4 initial groups (<30.0, 30.0-49.9, 50.0-74.9 and ≥75.0 nmol/L) based on the definition by the Institute of Medicine [7] and then combined the first 2 groups as <50.0 nmol/L to represent insufficient levels. The remaining 2 groups were combined as ≥50.0 nmol/L to represent sufficient levels.

### Vitamin D Single Nucleotide Polymorphisms and the Genetic Risk Score as Instrumental Variables

Extracted DNA from blood samples collected at HUNT2 or HUNT3 was stored in the HUNT Biobank. Genome-wide genotyping and imputation were carried out with sample and variant quality control by using Illumina Humina HumanCoreExome arrays [22]. We utilized 21 single nucleotide polymorphisms (SNPs) from 4 gene regions (GC, DHCR7, CYP2R1, and CYP24A1) as candidate instrument variables for serum 25(OH)D, as reported by Sofianopoulou et al [21]. These SNPs were selected based on their well-established biological roles in vitamin D transport, synthesis, and metabolism [21] and had strong associations with serum 25(OH)D levels (*P*-value < 5 × 10^−8^).

Information on 2 SNPs (rs139148694 and rs35870583) was missing from the HUNT Study because they did not pass imputation quality control (R^2^ of linkage disequilibrium >0.8). Consequently, 19 SNPs were used to construct an externally weighted genetic risk score (GRS) for our analyses. Using a GRS instead of individual genetic variants can ensure that a large proportion of serum 25(OH)D can be accounted for and therefore reduce weak instrument bias and increase statistical power [23]. The externally weighted GRS was calculated as the sum of the number of effect alleles carried for each SNP weighted by the reported β coefficient for serum 25(OH)D derived from the study by Sofianopoulou et al [21]. The characteristics of the 19 individual SNPs are shown in Table S1 [24].

### Other Baseline Variables

In HUNT3, body weight, height, and blood pressure were measured by health professionals at the clinical examination. Body mass index was calculated as weight in kilograms divided by height squared in meters (kg/m^2^). Other covariates included sex (women and men), smoking status with detailed pack-years information [never, former (<10, 10-20 and >20 pack-years), and current (<10, 10-20 and >20 pack-years)], alcohol consumption (never, 1-4, and ≥5 times/month), physical activity (inactive, low, moderate, and active), occupation status (class I-VII), hypertension (yes and no), coronary heart disease (yes and no), diabetes (yes and no), serum creatinine (µmol/L), and serum cholesterol (<5.2 nmol/L as desirable, 5.2-6.2 nmol/L as borderline, and >6.2 nmol/L as high). Hypertension was defined as systolic blood pressure ≥140 mmHg or diastolic blood pressure ≥90 mmHg or self-reported use of antihypertensive medication [25-27]. Coronary heart disease (CHD) was defined based on a “yes” answer to questions about angina pectoris or heart attack history. Diabetes was defined by a self-reported history and/or a nonfasting blood glucose level above 11 mmol/L. Missing information on each of the mentioned variables was included in the analyses as an “unknown” category. We used the same categorization of variables as in previous HUNT publications [12, 28].

### Verified AF Diagnosis

AF diagnoses were retrieved from discharge registers at all the regional hospitals in North Trøndelag County using the unique 11-digit personal identification numbers. The diagnosis was based on the International Classification of Diseases, Tenth Revision code I48 for AF/atrial flutter [29]. The verified AF diagnosis was AF or atrial flutter confirmed by a physician after reviewing the electrocardiograms according to standard criteria [1]. To confirm AF cases among participants with physician-diagnosed AF but no electrocardiogram information, 2 physicians independently assessed the available data, and the “probable AF” cases agreed by both physicians were treated as verified AF [29]. Persons who only had an episode of AF within the first 7 days after cardiac surgery, during the acute phase of a myocardial infarction, or during episodes of severe hemodynamic instability (eg, sepsis or major noncardiac surgery) were not regarded as having incident AF [29].

### Statistical Analysis

Baseline characteristics were presented for participants in the prospective cohort study (n = 3394) and the 1-sample MR study (n = 36 554). The *P*-value for the test of linearity was less than .05, which indicated that the association between the serum 25(OH)D levels and the incidence of AF might have deviated from a linear association. Thus, we only treated serum 25(OH)D levels as a categorical variable in the analysis. Cox proportional hazard models were used to examine the potential associations. We assessed the proportional hazards assumption by Schoenfeld residuals for exposure and all covariates. The results suggested that the proportional hazard assumption held for both exposure and covariates. Crude and adjusted hazard ratios (HRs) with 95% CIs were calculated with age as the underlying time scale. Potential confounders were selected based on previous knowledge [30-32] and directed acyclic graph. In the main model, we adjusted for sex, body mass index, smoking status, alcohol consumption, physical activity, occupation, hypertension, and CHD. Diabetes, serum creatinine, and serum cholesterol status were adjusted in an additional model since they may be mediators of the associations [31, 32].

To test the robustness of the findings in the prospective cohort analysis, we performed a series of additional analyses: (1) We classified the serum 25(OH)D levels into categories (<30.0, 30.0-49.9, 50.0-74.9, and ≥75.0 mmol/L) [7] to gain a more detailed understanding between serum 25(OH)D and AF. (2) We excluded the first 3-year follow-up and participants with CHD at baseline to address reverse causality due to existing but undiagnosed AF. (3) We conducted multivariable chained imputation with fully conditional specification (m = 10 imputed datasets) for missing information of all covariates to address residual confounding. (4) We performed a positive outcome control analysis using diabetes as alternative outcome [33] in the prospective cohort population to investigate potential selection bias [34]. Meta-analyses of observational studies and RCTs have consistently suggested an inverse association between serum 25(OH)D and diabetes risk [35]. If we observe the same direction of association, selection bias may not be a major methodological issue in our population. (5) We performed a competing risk analysis based on the Fine-Gray model to address possible competing risk due to death [36].

Furthermore, we conducted a 1-sample MR analysis to assess the potential causal association between genetically predicted serum 25(OH)D levels (per 10 nmol/L decrease) and the incidence of AF. A Wald ratio method was applied to compute the MR estimates [15, 37]. We computed a MR-driven HR by applying the natural exponential function of the ratio of coefficients. All regression models were adjusted for age, sex, batch, and 20 principal components [21].

In the sensitivity analysis of the 1-sample MR approach, we first tested the relevance assumption using the F statistic and R^2^ value for the association between the GRS and serum 25(OH)D levels in the subcohort of 3469 individuals. The GRS is regarded as an adequate instrument variable if the *F*-statistic >10 [38]. Next, we tested the associations between the GRS and the available confounders among the 36 554 individuals using linear or logistic regressions. The GRS should not be associated with any potential confounders according to the independence assumption. We further assessed the exclusion assumption using SNP-based 2-sample methods such as the inverse-variance weighted method, MR-Egger, simple median, weighted median [39, 40], and Mendelian Randomization Pleiotropy RESidual Sum and Outlier (MR-PRESSO) [41] methods.

All the statistical analyses were performed with STATA/SE 16.1 (College Station, TX, USA) or R (4.3.2). The package “TwoSampleMR” was used for the SNP-based 2-sample methods in R.

All participants gave their informed written consent for participation in HUNT. The current study was approved by the Norwegian Regional Committees for Medical and Health Research Ethics (no. 434217).

## Results

Table 1 shows that the distributions of all baseline characteristics were similar in the prospective cohort (n = 3394), the subcohort for genetic instrument-exposure association (n = 3469) in the 1-sample MR, and the entire cohort for the genetic instrument-outcome association (n = 36 554).

**Table 1.** Baseline (HUNT3) characteristics of participants included in the prospective cohort and the 1-sample MR analyses

Variables	Cohort for prospective analysis	Subcohort for genetic instrument-exposure association in the one-sample MR	Cohort for genetic instrument-outcome association in the one-sample MR
Number of subjects	3394	3469	36 554
Age (years)	57.3 ± 13.2	57.3 ± 13.2	58.1 ± 13.3
Sex (women), %	55.5	54.7	55.2
Season-standardized 25(OH)D level (nmol/L)	53.7 ± 15.1	53.9 ± 15.1	–
BMI (kg/m^2^)	27.3 ± 4.3	27.3 ± 4.3	27.4 ± 4.3
Smoking status, % (never/former/current/unknown)	41.4/32.6/23.1/3.0	41.2/33.0/22.8/2.9	40.3/34.3/22.3/3.1
Alcohol consumption (times/month), % (never/1-4/≥5/unknown)	4.7/77.6/15.1/2.6	4.7/77.5/15.2/2.7	4.3/77.5/15.3/3.0
Physical activity, % (inactive*^a^*/active*^b^*/unknown)	8.0/45.9/46.1	8.0/45.7/46.3	8.1/44.7/47.2
Occupation in EGP,*^c^* % (I-III/IV-VII/unclassified)	61.3/34.6/4.1	60.9/34.9/4.2	61.7/33.6/4.7
Hypertension, % (no/yes)	55.2/44.8	50.7/49.2	49.5/50.5
Coronary heart disease, % (no/yes)	93.3/6.7	93.3/6.7	92.8/7.2
Diabetes, % (no/yes)	95.3/4.7	95.2/4.8	94.5/5.5
Serum creatinine (µmol/L)	67.8 ± 17.8	68.5 ± 19.0	68.5 ± 19.1
Serum cholesterol*^d^* (mmol/L), % (desirable/borderline/high/unknown)	34.2/37.0/27.1/1.7	34.2/36.7/27.1/1.6	34.4/37.1/26.3/2.9

Data are given as mean ± SD for continuous variables or percentage for categorized variables.

Abbreviations: 25(OH)D, 25-hydroxyvitamin D; BMI, body mass index; EGP, Erikson Goldthorpe Portocarero social class scheme; HUNT, Trøndelag Health Study; MR, Mendelian randomization.

^a^Inactive: no physical activity or only light physical activity ≤2 hours per week.

^b^Active: physical activity from low to high per week.

^c^Occupation was defined based on EGP social class scheme: EGP class I referred to administrative managers, politicians, or academic professions; class II referred to occupations with shorter college and university degrees; class III referred to office and customer service occupations, sales, service, and care professions; class IV referred to occupations in agriculture, forestry, and fishing; class V + VI referred to craftsmen, process and machine operators, or transport; class VII referred to occupations without education requirements.

^d^Desirable serum cholesterol referred to serum cholesterol less than 5.2 nmol/L, borderline referred to 5.2 to 6.2 nmol/L, and high was >6.2 nmol/L.

In the prospective cohort study, we first compared the baseline characteristics of adults across their serum 25(OH)D categories (Table S2) [24]. Compared with those with serum 25(OH)D levels ≥50.0 nmol/L, participants with serum 25(OH)D levels <50.0 nmol/L seemed to have a higher percentage of obesity, current smoking (>20 pack-years), hypertension, diabetes, and elevated cholesterol levels, as well as lower levels of moderate to high physical activity and lower percentage of occupational classes related to high social class. During a median follow-up of 12.1 years, 304 participants developed AF. Compared with serum 25(OH)D ≥ 50.0 nmol/L, serum 25(OH)D levels <50.0 nmol/L were associated with a 27% reduced incidence of AF after adjustment for confounders in the main model (HR 0.73, 95% CI 0.57-0.93) (Table 2). Additional adjustments for diabetes, serum creatinine, and cholesterol levels did not change the results.

**Table 2.** Prospective association of average serum 25(OH)D levels between HUNT2 and HUNT3 with the incidence of atrial fibrillation, the HUNT Study, 2006-2008 to 2021 (n = 3394)

			Crude*^a^*		Main model*^b^*		Additional model*^c^*	
	Cases	IR (per 1000 person-years)	HR	95% CI	HR	95% CI	HR	95% CI
Average serum 25(OH)D between HUNT2 and HUNT3	304							
<50.0 nmol/L	114	6.25	0.78	0.62-0.98	0.73	0.57-0.93	0.73	0.57-0.93
≥50.0 nmol/L	190	8.31	1.00	Reference	1.00	Reference	1.00	Reference

Abbreviations: 25(OH)D, 25-hydroxyvitamin D; CI, confidence interval; HR, hazard ratio; HUNT, Trøndelag Health Study; IR, incidence rate.

^a^Age was used as the time scale in the crude model.

^b^In the main model, we adjusted for sex, body mass index (as continuous), smoking [(never, former (<10, 10-20, and >20 pack-years), current (<10, 10-20, and >20 pack-years)], alcohol consumption, physical activity, occupation, hypertension, and coronary heart disease. Age was used as the time scale.

^c^Additional adjustment for diabetes, serum creatinine, and cholesterol status in addition to the covariates in the main model.

The results of additional analyses provided supportive evidence for our primary findings: (1) findings derived from 4 categories of serum 25(OH)D levels seemed to align with our primary results but with broader 95% CIs. However, the results did not demonstrate a linear dose-response association (Table S3) [24]. (2) Excluding the first 3 years follow-up and participants with CHD yielded similar results (Table S4) [24]. (3) Multiple imputation for all covariates with missing showed comparable association estimates (Table S5) [24]. (4) In the analysis using diabetes as a positive outcome control, serum 25(OH)D levels measured at HUNT2 were inversely associated with the risk of diabetes at HUNT3 (Table S6) [24], which is in line with the results of previous study [35]. (5) Competing risk due to death did not influence our primary results (Table S7) [24].

In the 1-sample MR, a genetically determined 10 nmol/L decrease in serum 25(OH)D was associated with a 7% reduction in AF incidence (HR 0.93, 95% CI 0.86 to 1.00) using the Wald ratio method (Table 3). Sensitivity analyses using SNPs-based 2-sample methods such as inverse-variance weighted method, MR-Egger, simple median, and weighted median methods generally supported our primary causal findings (Table 3).

**Table 3.** Associations of genetically predicted serum 25(OH)D levels (per 10 nmol/L decrease) with the incidence of atrial fibrillation in the 1-sample MR analysis (n = 36 554)

	Methods	HR	95% CI	*P-*value
GRS-based 1-sample MR analysis	Wald ratio	0.93	0.86-1.00	.04
SNPs-based 2-sample MR methods	IVW	0.92	0.85-0.98	.02
	MR-Egger	0.94	0.83-1.06	.31
	MR-Egger intercept	Effect size		
		0.01	−0.02-0.04	.63
	Simple median	0.89	0.77-1.04	.14
	Weighted median	0.94	0.87-1.02	.16

Abbreviations: 25(OH)D, 25-hydroxyvitamin D; CI, Confidence interval; GRS, genetic risk score; HR, hazard ratio; HUNT, Trøndelag Health Study; IVW, inverse-variance weighted method; MR, Mendelian randomization; MR-PRESSO, Mendelian Randomization Pleiotropy RESidual Sum and Outlier; SNP, single nucleotide polymorphism.

In addition, the GRS explained 8.6% of the variability in serum 25(OH)D levels among the subcohort of 3469 individuals, with an *F*-statistic of 324. The GRS did not appear to be associated with any of the measured confounders among the 36 554 individuals (Table S8) [24]. Cochran's Q tests suggested no heterogeneity (*P* for Q > .05, results not shown). The intercepts from the MR-Egger method did not deviate from 0, and the *P-*value for the intercept was .63 (Table 3). Thus, the MR-Egger method did not show evidence of a directional pleiotropic effect under the instrument strength independent of direct effect assumption [15]. No outliers were detected based on the results from the MR-PRESSO method.

## Discussion

We observed a positive association between serum 25(OH)D levels, representing the long-term average value over 10 years, and the incidence of AF after a median follow-up of 12 years in a prospective cohort study of the Norwegian adult population. Our 1-sample MR provided further evidence supporting this positive association.

Previous prospective studies did not suggest a clear association between serum 25(OH)D levels and the incidence of AF in the general population [10, 11]. However, most studies involved participants over 65 years [10], relied on a single serum 25(OH)D measurement, and did not verify AF cases [10, 11]. The measurement errors of exposure and outcome might give biased results. In our prospective study, we addressed these issues by using repeated measurements of serum 25(OH)D and verified AF diagnosis. This might be an explanation for the difference in findings.

We further found a positive association between a genetically determined 10 nmol/L decrease in the serum 25(OH)D levels and a reduced incidence of AF in our 1-sample MR. We need to interpret this finding cautiously, as no association was found in a previous 2-sample MR study that included a large number of AF cases (cases/controls = 60 620/970 216) [17]. However, that study used summary data for AF from 6 different cohorts, which may have introduced bias due to heterogeneity. Moreover, the 2-sample MR study relied on only 6 individual genetic variants as instruments, whereas we used an externally weighted GRS based on 19 vitamin D-related SNPs, potentially enhancing the instrument strength. In addition, we measured serum 25(OH)D twice, and the long-term average value may provide a more reliable estimate for the exposure and reduce the likelihood of type II errors.

Furthermore, mixed evidence on causality has been reported based on available RCTs with shorter follow-up durations [42, 43]. The Vitamin D and Omega-3 Trial Rhythm Study suggested no effect of vitamin D supplementation on the prevention of AF at 2000IU/day over 5 years among 25 119 healthy adults [42]. The included participants had relatively high baseline serum 25(OH)D levels of approximately 75 nmol/L. This might diminish the response to supplementation and subsequently lead to a null finding. In another Finnish RCT over 5 years among 2495 individuals, the authors found a potential benefit in preventing AF using a vitamin D supplementation dose similar to that used in the VIVAL Rhythm Study [43]. However, the beneficial effect was obtained from a post hoc analysis, and the small size of the RCT leaves room for chance findings.

Our study has several strengths. First, this is the first study using both the prospective cohort study and a 1-sample MR approach to investigate the potential association between serum 25(OH)D levels and AF incidence. Second, our study benefits from a relatively long follow-up duration over 12 years. Third, selection bias is likely minimal, as the HUNT Study had a relatively high participation rate compared with other studies [18]. Our study cohorts for the prospective analysis and the GRS-exposure association are fairly representative of the entire population used for the GRS-outcome association. In addition, our results using diabetes as a positive outcome control further confirmed that selection bias was not a major issue in this study. Fourth, measurement errors of exposure were better controlled by measuring serum 25(OH)D twice and using the average values to reflect its levels over time. The verified AF cases from the discharge registers demonstrated greater validity than those from the International Classification of Diseases codes alone [29]. Fifth, we had information on a panel of potential confounders at baseline, which could minimize confounding. Finally, using the 1-sample MR approach with individual-level data, we had the opportunity to investigate the 3 MR assumptions: (1) the instrument, composed of 19 SNPs with well-established biological roles in vitamin D transport, synthesis, and metabolism, explained 8.6% of the variability in serum 25(OH)D levels, suggesting its suitability as an appropriate instrumental variable; (2) we were able to investigate the associations between the GRS and a panel of potential confounders including lifestyle factors and clinical data; (3) there was no substantial violation of the exclusion assumption since we did not observe horizontal pleiotropy based on the results from the MR-Egger and MR-PRESSO methods, although pleiotropy could manifest in subtle ways.

Our study had several limitations. First, in our prospective cohort analysis, we cannot completely rule out the possibilities of residual confounding. The latest RCTs suggest that omega-3 supplements may be associated with an increased incidence of AF [44-46]. Since many Norwegians might obtain vitamin D supplementation through cod liver oil, which contains omega-3, omega-3 likely acted as a positive confounder in our observational analysis. However, the observed positive association between serum 25(OH)D and AF incidence remained unchanged after further adjustment for cod liver oil or omega-3 supplements (data not presented). Second, compared with the previous 2-sample MR study [17], the sample size in our 1-sample MR was smaller [17]. Nevertheless, our genetic instrument strength appeared to be stronger than the instrument strength in the 2-sample MR study (*F*-statistics: 324 in our study vs a range of 13-254 in previous studies). The discrepancy in findings underscores the need for more 1-sample MR studies with individual-level data and larger sample sizes in the future. Third, we were unable to investigate the potential causal nonlinear association between serum 25(OH)D and AF incidence. This is because 25(OH)D levels are not measured across the entire HUNT population and widely accepted methods for nonlinear MR analyses are currently not available [47]. Last, the HUNT population is a homogeneous population with over 97% Caucasians; the homogeneity may restrict the generalizability of the findings to other ethnic groups.

## Conclusion

We observed a positive association between long-term average serum 25(OH)D levels and the incidence of AF. The results from the 1-sample MR approach supported this association. More 1-sample MR studies with larger sample sizes are needed to clarify this association. Until then, clinical and public health recommendations regarding vitamin D supplementation for the prevention of AF risk should be made cautiously.

## Data Availability

Data from the HUNT Study that is used in research projects will, when reasonably requested by others, be made available on request to the HUNT Data Access Committee (hunt@medisin.ntnu.no). The HUNT data access information describes the policy regarding data availability (https://www.ntnu.edu/hunt/data).

## Abbreviations

25(OH)D: 25-hydroxyvitamin D
AF: atrial fibrillation
CHD: coronary heart disease
CI: confidence interval
GRS: genetic risk score
HR: hazard ratio
HUNT: Norwegian Trøndelag Health Study
MR: Mendelian randomization
MR-PRESSO: Mendelian Randomization Pleiotropy RESidual Sum and Outlier
PC: principal component
RCT: randomized controlled trial
SNP: single nucleotide polymorphism

## References

[1] Van Gelder IC, Rienstra M, Bunting KV, et al 2024 ESC guidelines for the management of atrial fibrillation developed in collaboration with the European association for cardio-thoracic surgery (EACTS): developed by the task force for the management of atrial fibrillation of the European Society of Cardiology (ESC), with the special contribution of the European heart rhythm association (EHRA) of the ESC. Endorsed by the European stroke organisation (ESO). Eur Heart J. 2024;45(36):3314‐3414. 39210723 10.1093/eurheartj/ehae176

[2] Stevens D, Harrison SL, Kolamunnage-Dona R, Lip GYH, Lane DA. The atrial fibrillation better care pathway for managing atrial fibrillation: a review. EP Europace. 2021;23(10):1511‐1527. 34125202 10.1093/europace/euab092 PMC8502499

[3] Wijesurendra RS, Casadei B. Mechanisms of atrial fibrillation. Heart. 2019;105(24):1860‐1867. 31444267 10.1136/heartjnl-2018-314267

[4] Basit S . Vitamin D in health and disease: a literature review. Br J Biomed Sci. 2013;70(4):161‐172. 24400428 10.1080/09674845.2013.11669951

[5] Cashman KD, van den Heuvel EG, Schoemaker RJ, Prévéraud DP, Macdonald HM, Arcot J. 25-Hydroxyvitamin D as a biomarker of vitamin D status and its modeling to inform strategies for prevention of vitamin D deficiency within the population. Adv Nutr. 2017;8(6):947‐957. 29141976 10.3945/an.117.015578 PMC5682995

[6] van Schoor NM, Lips P. Worldwide vitamin D status. Best Pract Res Clin Endocrinol Metab. 2011;25(4):671‐680. 21872807 10.1016/j.beem.2011.06.007

[7] Del Valle HB, Yaktine AL, Taylor CL, Ross AC. Dietary Reference Intakes for Calcium and Vitamin D. National Academies Press; 2011. 21796828

[8] Demir M, Uyan U, Melek M. The effects of vitamin D deficiency on atrial fibrillation. Clin Appl Thromb Hemost. 2014;20(1):98‐103. 22826443 10.1177/1076029612453762

[9] Patel D, Druck A, Hoppensteadt D, et al Relationship between 25-hydroxyvitamin D, renin, and collagen remodeling biomarkers in atrial fibrillation. Clin Appl Thromb Hemost. 2020;26:1076029619899702. 32072817 10.1177/1076029619899702 PMC7288844

[10] Ding X, Lai J, Zhang H, Guo Z. Vitamin D, vitamin D supplementation and atrial fibrillation risk in the general population: updated systematic review and meta-analysis of prospective studies. Front Nutr. 2023;10:1246359. 37810914 10.3389/fnut.2023.1246359 PMC10551443

[11] Alonso A, Misialek JR, Michos ED, et al Serum 25-hydroxyvitamin D and the incidence of atrial fibrillation: the atherosclerosis risk in communities (ARIC) study. Europace. 2016;18(8):1143‐1149. 26847078 10.1093/europace/euv395 PMC4974632

[12] Asante EO, Mai XM, Eldholm RS, et al Vitamin D status over time and cognitive function in Norwegian older adults: a prospective cohort of the HUNT study. J Nutr Health Aging. 2023;27(1):30‐37.36651484 10.1007/s12603-022-1867-8

[13] Hutcheon JA, Chiolero A, Hanley JA. Random measurement error and regression dilution bias. BMJ. 2010;340(jun23 2):c2289. 20573762 10.1136/bmj.c2289

[14] Rutter CE, Millard LAC, Borges MC, Lawlor DA. Exploring regression dilution bias using repeat measurements of 2858 variables in ≤49 000 UK biobank participants. Int J Epidemiol. 2023;52(5):1545‐1556. 37336529 10.1093/ije/dyad082 PMC10555784

[15] Sanderson E, Glymour MM, Holmes MV, et al Mendelian randomization. Nat Rev Methods Primers. 2022;2(1):6. 37325194 10.1038/s43586-021-00092-5 PMC7614635

[16] Lawlor DA, Harbord RM, Sterne JA, Timpson N, Davey Smith G. Mendelian randomization: using genes as instruments for making causal inferences in epidemiology. Stat Med. 2008;27(8):1133‐1163. 17886233 10.1002/sim.3034

[17] Zhang N, Wang Y, Chen Z, et al Circulating vitamin d concentrations and risk of atrial fibrillation: a Mendelian randomization study using non-deficient range summary statistics. Front Nutr. 2022;9:842392. 35782933 10.3389/fnut.2022.842392 PMC9248862

[18] Krokstad S, Langhammer A, Hveem K, et al Cohort profile: the HUNT study, Norway. Int J Epidemiol. 2013;42(4):968‐977. 22879362 10.1093/ije/dys095

[19] Åsvold BO, Langhammer A, Rehn TA, et al Cohort profile update: the HUNT study, Norway. Int J Epidemiol. 2023;52(1):e80‐e91. 35578897 10.1093/ije/dyac095 PMC9908054

[20] Næss M, Kvaløy K, Sørgjerd EP, et al Data resource profile: the HUNT biobank. Int J Epidemiol. 2024;53(3):dyae073. 38836303 10.1093/ije/dyae073 PMC11150882

[21] Sofianopoulou E, Kaptoge SK, Afzal S, et al Estimating dose-response relationships for vitamin D with coronary heart disease, stroke, and all-cause mortality: observational and Mendelian randomisation analyses. Lancet Diabetes Endocrinol. 2024;12(1):e2‐e11. 38048800 10.1016/S2213-8587(23)00287-5 PMC7615586

[22] Brumpton BM, Graham S, Surakka I, et al The HUNT study: a population-based cohort for genetic research. Cell Genom. 2022;2(10):100193. 36777998 10.1016/j.xgen.2022.100193 PMC9903730

[23] Burgess S, Thompson SG. Use of allele scores as instrumental variables for Mendelian randomization. J Epidemiol. 2013;42(4):1134‐1144. 10.1093/ije/dyt093 PMC3780999 24062299

[24] Jiang L, Sun Y-Q, Malmo V, Mai X-M. 2025. Supplementary tables for “Serum vitamin D levels over time and the incidence of atrial fibrillation in The HUNT Study”. Zenodo. 10.5281/zenodo.17448560. Deposited 26 Nov 2025. PMC12635478 41278013

[25] Stenehjem JS, Hjerkind KV, Nilsen TI. Adiposity, physical activity, and risk of hypertension: prospective data from the population-based HUNT study, Norway. J Hum Hypertens. 2018;32(4):278‐286. 29483587 10.1038/s41371-018-0042-5

[26] Bakris G, Ali W, Parati G. ACC/AHA versus ESC/ESH on hypertension guidelines. J Am Coll Cardiol. 2019;73(23):3018‐3026. 31196460 10.1016/j.jacc.2019.03.507

[27] Vimaleswaran KS, Cavadino A, Berry DJ, et al Association of vitamin D status with arterial blood pressure and hypertension risk: a Mendelian randomisation study. Lancet Diabetes Endocrinol. 2014;2(9):719‐729. 24974252 10.1016/S2213-8587(14)70113-5 PMC4582411

[28] Jiang L, Sun Y-Q, Brumpton BM, Langhammer A, Chen Y, Mai X-M. Body mass index and incidence of lung cancer in the HUNT study: using observational and Mendelian randomization approaches. BMC Cancer. 2022;22(1):1152. 36348315 10.1186/s12885-022-10215-0 PMC9644519

[29] Malmo V, Langhammer A, Bonaa KH, Loennechen JP. Ellekjaer H: validation of self-reported and hospital-diagnosed atrial fibrillation: the HUNT study. Clin Epidemiol. 2016;8:185‐193. 27354826 10.2147/CLEP.S103346 PMC4910677

[30] Wang Q, Richardson TG, Sanderson E, Tudball MJ, Ala-Korpela M, Davey Smith G. Holmes MV: a phenome-wide bidirectional Mendelian randomization analysis of atrial fibrillation. Int J Epidemiol. 2022;51(4):1153‐1166. 35292824 10.1093/ije/dyac041 PMC9365635

[31] Sun YQ, Langhammer A, Wu C, et al Associations of serum 25-hydroxyvitamin D level with incidence of lung cancer and histologic types in Norwegian adults: a case-cohort analysis of the HUNT study. Eur J Epidemiol. 2018;33(1):67‐77. 29080012 10.1007/s10654-017-0324-1

[32] Garnvik LE, Malmo V, Janszky I, Wisløff U, Loennechen JP. Nes BM: physical activity modifies the risk of atrial fibrillation in obese individuals: the HUNT3 study. Eur J Prev Cardiol. 2020;25(15):1646‐1652. 10.1177/2047487318784365 29939081

[33] Denos M, Mai X-M, Åsvold BO, Sørgjerd EP, Chen Y, Sun Y-Q. Vitamin D status and risk of type 2 diabetes in the Norwegian HUNT cohort study: does family history or genetic predisposition modify the association? BMJ Open Diabetes Res Care. 2021;9(1):e001948. 10.1136/bmjdrc-2020-001948 PMC7786796 33402338

[34] Dusetzina SB, Brookhart MA, Maciejewski ML. Control outcomes and exposures for improving internal validity of nonrandomized studies. Health Serv Res. 2015;50(5):1432‐1451. 25598384 10.1111/1475-6773.12279 PMC4600355

[35] Pittas AG, Jorde R, Kawahara T. Dawson-hughes B: vitamin D supplementation for prevention of type 2 diabetes mellitus: To D or Not to D? J Clin Endocrinol Metab. 2020;105(12):3721‐3733. 32844212 10.1210/clinem/dgaa594 PMC7571449

[36] Austin P, Fine J. Practical recommendations for reporting fine-gray model analyses for competing risk data. Stat Med. 2017;36(27):4391‐4400. 28913837 10.1002/sim.7501 PMC5698744

[37] Palmer TM, Sterne JA, Harbord RM, et al Instrumental variable estimation of causal risk ratios and causal odds ratios in Mendelian randomization analyses. Am J Epidemiol. 2011;173(12):1392‐1403. 21555716 10.1093/aje/kwr026

[38] Davies NM, Holmes MV, Davey Smith G. Reading Mendelian randomisation studies: a guide, glossary, and checklist for clinicians. BMJ. 2018;362:k601. 30002074 10.1136/bmj.k601 PMC6041728

[39] Bowden J, Davey Smith G, Burgess S. Mendelian randomization with invalid instruments: effect estimation and bias detection through Egger regression. Int J Epidemiol. 2015;44(2):512‐525. 26050253 10.1093/ije/dyv080 PMC4469799

[40] Bowden J, Davey Smith G, Haycock PC, Burgess S. Consistent estimation in Mendelian randomization with some invalid instruments using a weighted median estimator. Genet Epidemiol. 2016;40(4):304‐314. 27061298 10.1002/gepi.21965 PMC4849733

[41] Verbanck M, Chen CY, Neale B, Do R. Detection of widespread horizontal pleiotropy in causal relationships inferred from Mendelian randomization between complex traits and diseases. Nat Genet. 2018;50(5):693‐698. 29686387 10.1038/s41588-018-0099-7 PMC6083837

[42] Albert CM, Cook NR, Pester J, et al Effect of marine omega-3 fatty acid and vitamin D supplementation on incident atrial fibrillation: a randomized clinical trial. JAMA. 2021;325(11):1061‐1073. 33724323 10.1001/jama.2021.1489 PMC7967086

[43] Virtanen JK, Hantunen S, Lamberg-Allardt C, et al The effect of vitamin D(3) supplementation on atrial fibrillation in generally healthy men and women: the Finnish vitamin D trial. Am Heart J. 2023;264:177‐182. 37302737 10.1016/j.ahj.2023.05.024

[44] Gencer B, Djousse L, Al-Ramady OT, Cook NR, Manson JE, Albert CM. Effect of long-term marine ɷ-3 fatty acids supplementation on the risk of atrial fibrillation in randomized controlled trials of cardiovascular outcomes: a systematic review and meta-analysis. Circulation. 2021;144(25):1981‐1990. 34612056 10.1161/CIRCULATIONAHA.121.055654 PMC9109217

[45] Nicholls SJ, Lincoff AM, Garcia M, et al Effect of high-dose Omega-3 fatty acids vs corn oil on major adverse cardiovascular events in patients at high cardiovascular risk: the STRENGTH randomized clinical trial. JAMA. 2020;324(22):2268‐2280. 33190147 10.1001/jama.2020.22258 PMC7667577

[46] Curfman G . Omega-3 fatty acids and atrial fibrillation. JAMA. 2021;325(11):1063. 33724309 10.1001/jama.2021.2909

[47] Hamilton FW, Hughes DA, Spiller W, Tilling K, Davey Smith G. Non-linear Mendelian randomization: detection of biases using negative controls with a focus on BMI, vitamin D and LDL cholesterol. Eur J Epidemiol. 2024;39(5):451‐465. 38789826 10.1007/s10654-024-01113-9 PMC11219394

